# Tight clustering for large datasets with an application to gene expression data

**DOI:** 10.1038/s41598-019-39459-w

**Published:** 2019-02-28

**Authors:** Bikram Karmakar, Sarmistha Das, Sohom Bhattacharya, Rohan Sarkar, Indranil Mukhopadhyay

**Affiliations:** 1 Department of Statistics, University of Pennsylvania, Philadelphia, PA USA; 2 Human Genetics Unit, Indian Statistical Institute, Kolkata, India

## Abstract

This article proposes a practical and scalable version of the tight clustering algorithm. The tight clustering algorithm provides tight and stable relevant clusters as output while leaving a set of points as noise or scattered points, that would not go into any cluster. However, the computational limitation to achieve this precise target of tight clusters prohibits it from being used for large microarray gene expression data or any other large data set, which are common nowadays. We propose a pragmatic and scalable version of the tight clustering method that is applicable to data sets of very large size and deduce the properties of the proposed algorithm. We validate our algorithm with extensive simulation study and multiple real data analyses including analysis of real data on gene expression.

## Introduction

Cluster analysis is one of the most important tools for empirical data analysis. The intuitiveness of the goal of a cluster analysis–grouping objects which are similar–is the reason why researchers from different fields tend to introduce various, and often similar, clustering algorithms as per their requirements. From the perspective of its ease of use, interpretability and adaptability to different scenarios, a few clustering methods stand out. Notable such methods are Hierarchical clustering^1^, *K*-means^2^, PAM^3^, self-organizing maps (SOM)^4,5^ and tight clustering^6^. The last one in this list, tight clustering, is more recent introduction to scientific practice and have received notable appreciation in the field of interest of the present article–microarray gene expression analysis. Tight clustering algorithm allows noisy observations and yields relatively small clusters in the order of their tightness. Both features promote the tight clustering algorithm well as a clustering algorithm for microarray data.

The study of microarray gene expression data is vital in the research for transcriptional activities in a biological organism. Gene clustering works as an essential intermediary tool in such studies by providing set of expression profiles that are common among themselves and different in between. The result of gene clustering largely helps the later step in microarray analysis of understanding co-regulation or scrutinizing specific pathways. A gene clustering algorithm is presented with the challenge of picking out the relevant small collection of genes out of a huge pool of genes that a practitioner can investigate further. Moreover, it is natural to believe that some genes may not belong to any cluster or biologically functional category that has some relevance with the present objective. Tight clustering algorithm is a perfect candidate for this job. Tight clustering is specifically advantageous because it provides tight and stable clusters in succession. Achieving this challenging goal requires several of repeated subsamplings. Underneath this bunch of resamplings, tight clustering method relies on *K*-means clustering algorithm. Thalamuthu *et al*.^7^ established that tight clustering consistently outperforms other clustering methods. Since then, new algorithms are being proposed in the line of the philosophy of tight clustering. To mention a few: penalised *K*-means approach can incorporate prior information while forming the clusters^8^, partially mixture model in clustering time-course gene expression data^9^, and Bayesian version of the algorithm^10^.

Although the tight clustering method is an intelligent algorithm that provides a reliable outcome in gene clustering, it fails to incorporate huge data of tens of thousands of gene expressions. These huge data sets, which are results of advances in biomedical imaging technologies and gene mapping technologies, allow us to simultaneously monitor a large number of genes or features and gives the opportunity to study interactions in biological regulatory processes. Unfortunately, tight clustering algorithm fails considerably, due to its computation heavy nature, for data sizes larger than 3000. The heavy burden of repeated use of *K*-means algorithm on many subsamples is unavoidable if a gene clustering algorithm is to live up to its challenge. Thus, a novel algorithm is required to adapt tight clustering algorithm for large gene expression data sets or other large data sets.

Our goal in this article is to provide a stable algorithm that allows the use of tight clustering in large data sets. The proposed algorithm follows the broader theme of splitting a problem into smaller problems and then combining the results of those smaller problems to arrive at a solution to the problem. The principle adopted here has received scattered recognition in broader literature^11^. Our algorithm takes advantage of various nice aspects of the original tight clustering algorithm. We present a complete analysis of the proposed algorithm along with time complexity analysis and probabilistic consistency.

The detailed methodology of our algorithm, that is developed below, starts with a brief recall of the traditional tight clustering idea. Then we propose our tight clustering algorithm for large datasets and gradually deduce the theoretical analyses for the same. The Results section examines the performance of the algorithm using simulation and real data sets while the Discussion section guides the practical use of the algorithm. Especially, we provide a clear guidance for the choice of parameters of the algorithm. We have written an R code to implement the proposed algorithm.

## Methods

Like tight clustering algorithm, we shall allow our proposed algorithm to return stable clusters in the decreasing order of tightness. Following the accepted strategy of analysing large data, we propose a resampling-based approach. The steps of the algorithm are informally discussed here.

The algorithm picks one cluster in each iteration. The first iteration begins with partitioning the data on multiple resamples. For each resample, it partitions the data set into small manageable partitions and extracts the tight-most cluster from each of them. After collecting these selected clusters from the partitions into an empty bucket, the distinct clusters are identified as candidate clusters based on a measure of distance between clusters in the bucket. This process uses a function *π* that decides whether two sets of profiles are similar. The tight-most cluster, from the list of candidate clusters, is identified using a suitable measure of dispersion *σ*. Data points of this resultant cluster are then removed from the original data set and remaining data set is fed into the next step of iteration to pull out the next cluster. But this time the bucket may already have those rejected clusters from the earlier iteration. This procedure is repeated until the required number of clusters are extracted. In the following we describe the methodology.

Suppose *X* is the data matrix with *n* rows and *d* columns. In gene clustering we want to extract disjoint subsets of *n* genes, say *C*^(1)^, *C*^(2)^, $\cdots$, *C*^(*k*)^, so that expression profile in one of these subsets differs from the general profile of other groups. Let the size of the *i* th subset be *n*_*i*_. Tight clustering algorithm ensures that each of these clusters is stable and extracted in order of their tightness, i.e., *C*^(1)^ is the tight-most cluster, then *C*^(2)^ is the next tight-most one, and so on, under the constraint that ${\sum }_{i=1}^{k}\,{n}_{i}\le n$. The strict inequality in sum of *n*_*i*_’s suggests the presence of noise in the data set.

### Tight clustering

In this section we summarise the tight clustering algorithm following Tseng and Wong^6^. Given a microarray data matrix of *n* genes, the tight clustering algorithm runs a sub-algorithm based on a number of consecutive choices of *k* in a *K*-means algorithm. For each of these different choices of *k*, it chooses the possible candidates for the tight-most cluster based on *K*-means on several sub-sampling of the *n* genes. In the following step the algorithm picks the stable one, thus extracting the first stable and tight set of genes that are likely to belong to a functional category. The second iteration for extracting the next set of similar gene patterns repeats the method on the remaining set of genes. This iterative method protects against presence of a large number of scattered genes and picks up a cluster of only relevant 10–50 genes that can be considered for further investigation. *K*-means algorithm is used as a convenience for the implementation of tight clustering, while other algorithms can be used. The local minima problem of *K*-means algorithm is addressed in this algorithm with a hierarchical sub-clustering.

The primary benefits of the tight clustering algorithm are listed below:

- The Tight clustering method addresses the local minimum problem of *K*-means clustering.
- The algorithm always ensures stable clusters and the extracted set of patterns are ordered according to their tightness.
- With the relaxed constraint ${\sum }_{i=1}^{k}\,{n}_{i}\le n$, the choice of the number of similar gene expression pattern becomes irrelevant.

These three characteristics make tight clustering algorithm useful in gene clustering^7^. To achieve a scaled version of tight clustering we ensure that the resulting algorithm also possesses these three properties.

### Extension of the tight clustering algorithm for large data sets

#### Partitioning

We randomly partition *n* gene data into *L*(*n*) parts. If *M* is a manageable size to run the original tight clustering algorithm, then we partition the data into $L(n)\sim \lceil \frac{n}{M}\rceil$ parts. To reduce the error due to partitioning this procedure is repeated *R*(*n*) times so that we are left with *M*_0_ = *R*(*n*) × *L*(*n*) manageable data sets. We extract tight-most cluster from each of these *M*_0_ data sets and store them in an empty bucket ${\mathcal B}$. The elements in this bucket can be enumerated as

$$ {\mathcal B} =\{{C}_{1},{C}_{2},\cdots ,{C}_{{M}_{0}}\}.$$

Elements of the bucket ${\mathcal B}$ are not necessarily disjoint, and they may not contain all the gene expressions. An element of the bucket simply tells us that the gene expressions therein are of similar pattern. The next step of the algorithm performs some operations on this bucket and redefines its members so that the gene expressions of each element will now likely have similar profile and gene expressions from different members will likely have significantly different profiles.

#### Combining

Consider the first member of the bucket, *C*_1_. We combine all the members of the bucket with pattern similar to that of *C*_1_ and redefine it with the new collection. Let us consider a decision function $\pi (\,\cdot \,,\,\cdot \,)$ that maps 2^*X*^ × 2^*X*^ to {0, 1}, where 2^*X*^ denotes the power set of *X*. If $\pi (C,\hat{C})=1$, we decide that the subsets *C* and $\hat{C}$ are of similar profile and otherwise they are of different profiles. Using this decision function, bucket elements are redefined as

1 $${C}_{1}:={C}_{1}\cup (\,{\cup }_{j=2}^{{M}_{0}} {\mathcal I} \,({C}_{j},\pi ({C}_{1},{C}_{j}))),$$

2 $${\rm{and}}\,{C}_{j}:= {\mathcal I} ({C}_{j},1-\pi ({C}_{1},{C}_{j}))\,for\,j=2,3,\ldots ,{M}_{0}.$$

Here, ${\mathcal I} (\,\cdot \,,\,\cdot \,)$ is a set valued function defined on 2^*X*^ × {0, 1} such that ${\mathcal I} (C,1)=C$ and ${\mathcal I} (C,0)=\varnothing$ (the empty set). At the end of this step, we are left with *M*_0_ members in bucket but now possibly some of them are empty. This process of redefining is carried out sequentially for *i* = 2, 3, …, *M*_0_ − 1

3 $${C}_{i}:={C}_{i}\cup (\,{\cup }_{j=i+1}^{{M}_{0}} {\mathcal I} ({C}_{j},\pi ({C}_{i},{C}_{j}))),$$

4 $${\rm{and}}\,{C}_{j}:= {\mathcal I} ({C}_{j},1-\pi ({C}_{i},{C}_{j}))\,for\,j=i+1,\ldots ,{M}_{0}.$$

A necessary constraint on the decision function *π* is that *π*(*ϕ*, *C*) = *π*(*C*, *ϕ*) = 0 for any $C\subset X$. Choices of the decision function *π* are discussed in the following section.

An element of the resultant bucket ${\mathcal B}$ is now characterized as either a null collection or a collection of gene expressions which are similar in themselves and different from other patterns. We can denote this method as an operation on ${\mathcal B}$, say ${\mathcal B} \,:\,={\mathbb{C}}( {\mathcal B} )$. In a sense of generality we note that, the above method, specified by the decision function *π*, is one of many possible methods that can be adopted to operate on ${\mathcal B}$ and redefine its members as having the required characteristics. Since the method of combining and then identifying distinct collection is carried out on the bucket, data points (or gene expressions) not belonging to any element of the bucket are not involved in this process.

#### Tight-most cluster

Finally, to obtain the tight-most cluster from the possible choices, i.e., the redefined members of ${\mathcal B}$, we require a measure of tightness of a set of data points. Suppose *σ*(*C*) is a positive valued function defined on non-null subsets of *X* that tells us how disperse the set of data points in *C* is. Then our desired cluster will be

5 $${C}^{(1)}\,:\,={\rm{\arg }}\,\mathop{{\rm{\min }}}\limits_{C\in  {\mathcal B} ,C\ne \varphi }\sigma (C).$$

The stability of this cluster is assured as each output of the tight clustering method ensures stable cluster.

#### Next iteration

After we obtain the tight-most cluster from the data, to get to the next one in line, we remove all data points of the obtained cluster from the whole data as well as from members of the bucket. Thus we are left with the pair $(X\backslash {C}^{(1)}, {\mathcal B} :=\{B\backslash {C}^{(1)}|B\in  {\mathcal B} \})$.

The remaining data points are partitioned and tight-most cluster of each of these parts is put into the bucket ${\mathcal B}$. Notice that, unlike at the initiation of the algorithm we possibly already have nonempty collections in the bucket. The operation ${\mathbb{C}}$ is carried out on the new bucket and afterwards *C*^(2)^ is obtained by comparing dispersion measure of the members of ${\mathcal B}$.

#### Output

Repeating the process above we can extract *k* clusters of the data, $({C}^{(1)},{C}^{(2)},\cdots ,{C}^{(k)})$ of sizes ${n}_{1},{n}_{2},\cdots ,{n}_{k}$ (say) respectively. This extension of tight clustering algorithm retains the properties of the original algorithm. Specifically, ${\sum }_{i=1}^{k}\,{n}_{i}\le n$, i.e., it still allows for the noise in data. In large number of genes, we believe that several of them will be noise points. Thus, contamination due to them is more important problem than in a small number of genes. It is also important to note that even if one aims to obtain *k* clusters the algorithm allows for the possibility that *n*_*i*_ is 0 after a certain *k*_0_ < *k*. Thus, in case the data do not contain any noise, with a large value of *k* we expect to obtain all the clusters in the data.

The proposed algorithm has three tuning parameters as its input, *π*, *σ*, and *R*(·). We discuss below the choices of these parameters in practical implementation.

### The decision function *π*: 2^*X*^ × 2^*X*^ → {0, 1}

The value of the function $\pi (C,\hat{C})$ should indicate whether the subsets *C* and $\hat{C}$ are close enough to be treated as a single cluster of data points. An acceptable parametric form of the function would be given by

6 $$\pi (C,\hat{C})={\mathbb{I}}(d(C,\hat{C}) < \gamma ),$$

where $d(C,\hat{C})$ measures the distance between *C* and $\hat{C}$; *γ* is a thresholding parameter. One reasonable choice of *d* is

7 $$d(C,\hat{C})=\frac{{{\rm{avg}}}_{x\in C,x^{\prime} \in \hat{C}}(dist(x,x^{\prime} ))}{{{\rm{\max }}}_{x\in C,x^{\prime} \in \hat{C}}(dist(x,x^{\prime} ))},$$

and $d(C,\hat{C})=\infty$ if at least one of *C* or $\hat{C}$ is empty. Other possible choices like Ward’s variance distance between two clusters^12^, which is widely applied in hierarchical clustering, can be used.

Another possible choice of the function *π*, not dependent on any metric structure, is based on minimum spanning tree (mst), which only makes use of the point data cloud structure. Let *d*_*mst*_(·) denote the maximum edge length of the mst based on a collection of data points. We set $\pi (C,\hat{C})={\mathbb{I}}(2{d}_{mst}(C\cup \hat{C}) > {d}_{mst}(C)+{d}_{mst}(\hat{C}))$. This decision function is particularly appealing to our problem. The function *d*_*mst*_(·) gives an idea of the spread of a collection of data points in the sample space thus incorporating distance between any two points. This is a well-studied object with various appealing properties^13^ and has also been used for clustering algorithms^14,15^. For two distinct tight clusters, naturally the maximum edge length of the combined clusters should always be much larger than that for individual tight clusters whereas they would be almost same if the two clusters are situated not far from each other. Results of this article are based on this choice of *π*.

### Measure of tightness ${\boldsymbol{\sigma }}\,:\,{{\bf{2}}}^{{\boldsymbol{X}}}\to {{\mathbb{R}}}^{{\boldsymbol{+}}}$

For a collection of data points *C*, *σ*(*C*) should in some sense measure the tightness of *C*. Smaller the value of *σ*(*C*), the tighter would be the set *C*. The obvious required properties of *σ* would be translation and rotation invariance (i.e., *σ*(*α* + *C*) = *σ*(*C*) and *σ*(*UC*) = *σ*(*C*), where $C\subset X$, *α* is any vector, and *U* is any unitary matrix. Here *α* + *C* = {*α* + *x*: *x* ∈ *C*} and similarly *UC* is defined.) Below are some of the choices for this function

- *σ*(*C*) = *tr* (*Var*(*C*)).
- *σ*(*C*) = maximum eigenvalue of *Var*(*C*).
- *σ*(*C*) = product of non-zero eigenvalues of *Var*(*C*).
- *σ*(*C*) = maximum pairwise distance between points in *C*.
- *σ*(*C*) = average edge length of spanning tree based on *C*.

These choices are only some of the popular measures used in the literature to measure dispersion of a collection of data points in high dimension. Among various possible choices of functions *π* and *σ*, the ones used in a given practical study must be justified based on the problem at hand. To avoid any subjectivity, we adopt the last one in the list for our results in the next section, mainly because we have used the decision function *π* based on minimum spanning tree. Naturally, span of the tree gives a nice idea about the tightness of the cluster.

### Time complexity

Large data algorithms ask for careful analysis of complexity of its run time. Below we provide a rough worst case bound on the time complexity of the overall algorithm. The complexity calculation is based on the standard notation used in Tseng and Wong^6^. We assume that the tight clustering routine uses parameters, *B* = total number of resampling to obtain co-membership matrix, *k*_0_ = minimum number of clusterings to be fed into *K*-means sub-routine, and *m* = maximum number of subsequent *K*-means sub-routine algorithm called to get stable clusters.

**Theorem**: With a data set of size *n* and a bucket ${\mathcal B}$ of *N* data points, if we adopt mst based functions *π* and *σ*, then run time of extracting the tight-most cluster is bounded by

8 $$R(n)L(n)B\cdot O({\lfloor \frac{n}{L(n)}\rfloor }^{d({k}_{0}+m)+1})+{(N+L(n)R(n))}^{2}\cdot O((n+N)\,\mathrm{log}\,(N+n)).$$

**Proof**: Following Theorem 3 of Inaba *et al*.^16^, the run time of the clustering sub-algorithm is bounded by

9 $$R(n)L(n)B\times O(\sum _{i=0}^{m}\,{\lfloor \frac{n}{L(n)}\rfloor }^{d({k}_{0}+i)+1}),$$

which is exactly the first expression of the complexity bound in our statement of the theorem.

The combining operator ${\mathbb{C}}$ now acts on sub-collection of data points of at most (*N* + *n*) points. Borrowing results from March *et al*.^17^ we see that the run time of the operation ${\mathbb{C}}$ is of the order

10 $${(N+R(n)L(n))}^{2}O((N+n)\,\mathrm{log}\,(N+n)).$$

Finally, the operations of the *σ* function on output of operator ${\mathbb{C}}$ will run in

11 $$(N+R(n)L(n))O((N+n)\,\mathrm{log}\,(N+n)),$$

time, which is dominated by the run time of the combining sub-algorithm. Proof of the theorem follows by summing up these complexity calculations. ▯

The discussion of choice of *R*(*n*) is discussed elsewhere. Note that if we choose *R*(*n*) as indicated there, then *R*(*n*) = *O*(*L*(*n*)^−1^) and *L*(*n*) = *O*(*n*). Thus if we start with an empty bucket (i.e., *N* = 0), the proposed large data implementation of the tight clustering algorithm has the same order (*O*(*n* log (*n*))) of time complexity as the usual tight clustering algorithm with same choices of the parameters of the algorithms. But later iterations will have higher time complexity.

### Probability of concordance

In this section we prove that the proposed method is consistent in the sense that the probability of concordance goes to 1 as the number of repetitions is increased to infinity. By the term ‘probability of concordance’ we mean the probability of the event of two data points *x*_1_, *x*_2_ originally belonging to the tight-most cluster (${\mathscr{C}}$) in the data set *X* will be clustered together in the tight-most cluster through our proposed method.

For our calculations we assume that in each partition of the data where either of the points *x*_1_, *x*_2_ fall, the tight clustering algorithm picks up a subset of ${\mathscr{C}}$ containing both points. This assumption simply tells that original tight clustering method is doing the job it was assigned to do. Then the probability of concordance is given by the probability of the complement of the event that, *x*_1_, *x*_2_ falls into different partitions for each repetition and the operator ${\mathbb{C}}$ fails to combine any of the cluster containing *x*_1_ with any of the clusters containing *x*_2_. Thus, the probability of concordance is

12 $$\begin{array}{l}1-{\{(\begin{array}{c}L(n)\\ 2\end{array})\times \frac{1}{L(n)(L(n)-\mathrm{1)}}\}}^{R(n)}\times \{{(1-{p}_{\pi }^{(1)})}^{R(n)(R(n)-\mathrm{1)}}+{(1-{p}_{\pi }^{(2)})}^{R(n)}\}\\ \,=\,1-\frac{1}{{2}^{R(n)}}\times \{{(1-{p}_{\pi }^{(1)})}^{R(n)(R(n)-1)}+{(1-{p}_{\pi }^{(2)})}^{R(n)}\},\end{array}$$

where ${p}_{\pi }^{(1)}$ is the probability that two random sample each of size less than or equal to $\lfloor \frac{n}{L(n)}\rfloor +1$ of ${\mathscr{C}}$ one containing *x*_1_ (say ${{\mathscr{C}}}_{1}$) and another containing *x*_2_ (say ${{\mathscr{C}}}_{2}$) fails to combine, i.e., $\pi ({{\mathscr{C}}}_{1},{{\mathscr{C}}}_{2})=0$, while ${p}_{\pi }^{(2)}$ is the probability of the same when ${{\mathscr{C}}}_{1}$ and ${{\mathscr{C}}}_{2}$ are necessarily disjoint. Both quantities are less than 1. Therefore, when the number of repetitions is increased to infinity, independently of the data, the probability of concordance increases to 1. Thus, our proposed algorithm can capture the true association between two points and this in turn proves the validity of our algorithm.

### Number of repetitions *R*(*n*)

As per the description of the algorithm, given that we have a data of size *n*, the number *L*(*n*) gives the number of equal (almost) random partition on which we should apply our tight clustering algorithm and *R*(*n*) gives the number of times we should repeat this to reduce the error due to partitioning.

As suggested earlier, if computational power allows us to run our basic tight clustering algorithm on a data of size *M*, we can take $L(n)\sim \lceil \frac{n}{M}\rceil$. After making a choice for *L*(*n*), we can argue a plausible choice of *R*(*n*) as follows.

Using this formula for the probability of concordance in the last section, we can give a simple lower bound for the probability of the event that the proposed algorithm provides concordance for all pair of points. In the probability of discordance formula, the first term is dominated by the second term. Thus, through a simple application of Boole’s inequality the probability of concordance for all the pair of data points is at least

13 $$1-\frac{1}{{2}^{R(n)}}\times \frac{{n}^{2}}{2}\times 2{(1-{p}_{\pi }^{(2)})}^{R(n)}.$$

We choose the value of *R*(*n*) such that this lower bound is more than some number fraction *p* close to 1. Though ${p}_{\pi }^{(2)}$ is not known, it is expected that ${p}_{\pi }^{(2)}$ is going to be a decreasing function of *n*/*L*(*n*). We assume that $1/{p}_{\pi }^{(2)}$ is a polynomial in *n*/*L*(*n*). In that case we make a conservative choice

14 $$R^{\prime} (n)\,:\,=\lceil \frac{\mathrm{log}\,(1-p)-2\,\mathrm{log}\,(n)}{-\,\mathrm{log}\,(2)+\,\mathrm{log}\,(1-1/(n/L(n)))}\rceil .$$

**Figure 1:**
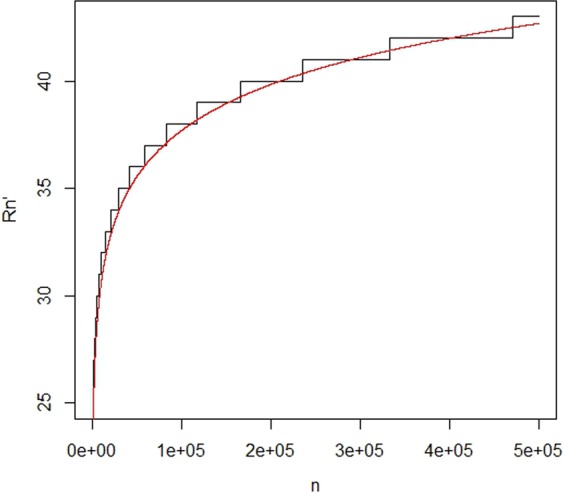
Plot for *R*′(*n*) as a function of *n*. In this plot we let *M* = 5000 and $L(n)=\lceil \frac{n}{M}\rceil$. *R*′(*n*) is suggested based on the requirement that probability of concordance is at least *p* = 0.95. The red curve is that of 2 + 3.1log(*n*).

Here we suggest *p* as 0.95 or more. Figure 1 gives a sense of the rate of *R*′(*n*).

In certain situations, this choice of *R*′(*n*) may not be practically feasible. Based on hardware availability there could be a restriction on the number of sub clustering operations, *R*′(*n*) × *L*(*n*), to be less than Λ (say), imposed by the computational power. In which scenario, our proposed choice of *R*(*n*) is

15 $$R(n)\leftarrow \,{\rm{\min }}\{R^{\prime} (n),\frac{{\rm{\Lambda }}}{L(n)}\}.$$

### Practical choice of *R*(*n*) in the presence of noise

The proposed algorithm uses two sub-algorithms, tight clustering and combining operator, both of which are prone to error. When the presence of noise is known in the data, these sub-algorithms come into play accumulating noise data on every repetition. With the increase in *R*(*n*) the performance of the algorithm becomes better up to a certain value of *R*(*n*). But after that, increase in *R*(*n*) would accumulate more noise thus reducing overall performance of the algorithm. Since noise points should not go into any cluster; it is randomly distributed over partitions and hence may go into some clusters which in effect reduces the performance substantially. This is evident from our detailed simulation study.

**Figure 2:**
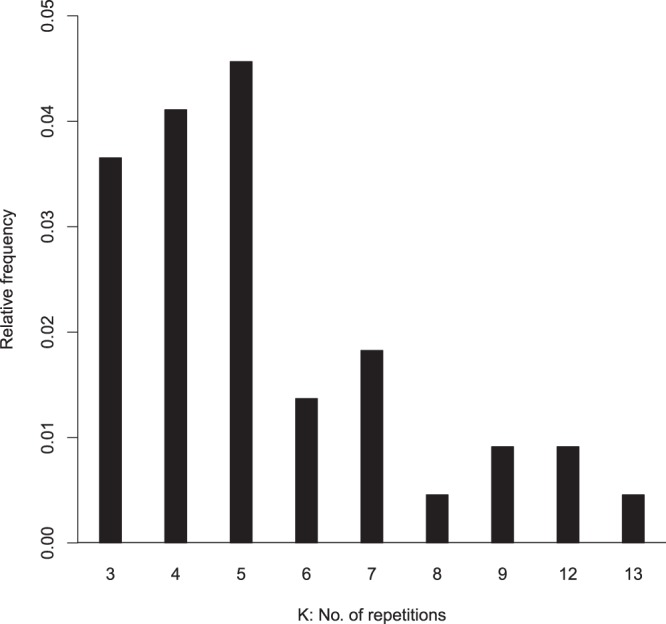
Frequency distribution of where Rand Index is maximum over repetitions.

The specifics of the simulation structure adopted is described in the next section. Figure 2 shows a frequency distribution of where maximum Rand index is attained when *R*(*n*) varies from 3 to 15, based on several simulation scenarios. The frequency initially increases showing better and better performance until it reaches maximum and then starts falling with increase in *R*(*n*). We observe that the difference in Rand index for a given data set between the maximum and the next one or two is small. We study this phenomenon using both these Rand indices (Supplementary Figs S1 and S2). For the sake of saving computation time we present the results in Results section for *R*(*n*) = 3. Although theoretically it seems that as *R*(*n*) goes to infinity, performance will only get better, but careful analysis suggests that it cannot go beyond a certain level. This strengthens our logic to apply the algorithm with relatively small number of repetitions.

## Results

### Simulation study

We simulate microarray data using the scheme mentioned in Thalamuthu *et al*.^7^ considering sample sizes to be 10000, 20000, 30000, 50000, and 100000 genes in each data set. Certain percentage of noise genes (e.g., 5%, 10%, 15%, and 20%) is also added to each data set. The dimension of the data sets considered are of 5, 10, 15, and 20 dimensions. Thus, we have considered wide range of scenarios with varying percentage of noise points, dimension and sample size. The sample size of any data set considered here exceeds far beyond the permissible range of existing tight cluster algorithm.

For each of the data sets we use *L*(*n*) = 2000 to implement our algorithm. Hence, the standard tight cluster sub-algorithm is applied to a data set having a maximum of 2000 points. An early simulation based analysis showed that the number of repetitions cannot be too large and we thus run it for values of *R*(*n*) ranging 3, 4, …, 7. Therefore, our simulations seem to be extensive enough on 600 data sets to provide a clear picture of the performance of the proposed algorithm. In each case we calculate the Rand index with noise and without noise and take a convex combination of these two measures to define a modified Rand index as described in Thalamuthu *et al*.^7^. Since the underlying true scenario is known for simulated data sets, this modified Rand index is used to measure the quality of the final clusters obtained through our algorithm.

**Figure 3:**
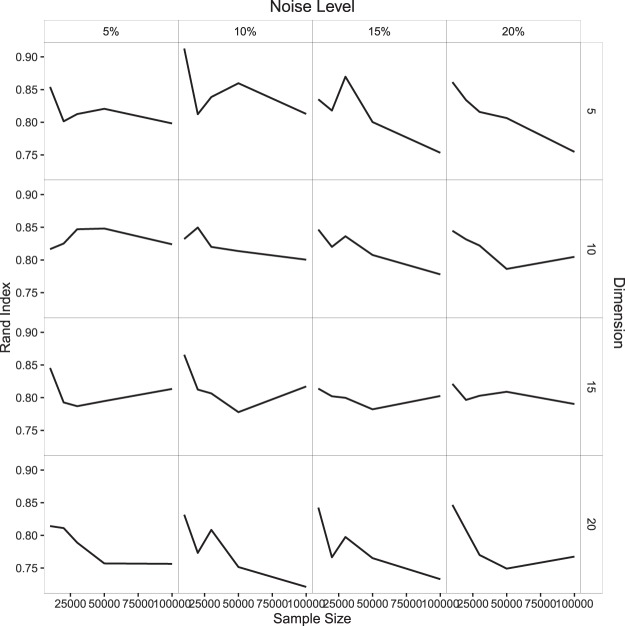
Average Rand index for different sample sizes, noise levels and dimensions.

Based on our data analysis, we observe that the performance of our proposed method, in applying the tight cluster algorithm, is very good as given by the modified Rand index. The results are summarised in Fig. 3 and Table 1. This Rand index is always very high and above 0.75 in almost all cases. This high value ensures the usefulness of our algorithm in clustering large data by tight clustering method. We observe that Rand index decreases slightly for data sets of size more than 50000; however, the noise level plays an important role. Figure 3 illustrates that if we have smaller dimension and less percentage of noise points, we get better result while for higher dimension more amount of noise is preferred. This is true over varying dimension and noise level, which is not counter intuitive. Although it seems that Rand index decreases with increase in dimension, but even with 20% noise this decrease is hardly noticeable for higher dimension.

**Table 1:** Average Rand index for simulated data varying sample size, number of clusters, and percentage of noise points (noise%).

*n*_*c*_		SS = 10000			SS = 20000		
		5	10	15	5	10	15
d	noise						
10	5%	0.843	0.876	0.875	0.777	0.838	0.840
	10%	0.820	0.856	0.868	0.811	0.855	0.833
	15%	0.836	0.821	0.857	0.798	0.817	0.830
	20%	0.863	0.823	0.865	0.830	0.839	0.839
	5%	0.790	0.832	0.856	0.709	0.813	0.834
20	10%	0.754	0.837	0.866	0.742	0.816	0.834
	15%	0.775	0.831	0.855	0.732	0.787	0.821
	20%	0.791	0.806	0.869	0.744	0.744	0.823

All values have standard deviations between 0.01–0.06. d = dimension, SS = sample size, *n*_*c*_ = number of clusters.

The proposed algorithm works as expected with relevant amount of noise in the data corresponding to the dimension (under the default parameter choices). Dimensionality is not an important influencing factor to affect the performance provided there is sufficient amount of noise. It is indeed true that with increase in dimension the data points tend to move away for each other resulting in a good number of scattered points that would be detected through the algorithm of tight clustering. Consequently, presence of noise would be natural in case of high dimensional data sets. Plots here give a clear picture to indicate the overall good performance of our proposed algorithm under various scenarios based on the amount of noise points, varying dimensions and sample sizes (see also Supplementary Figs S3–S15).

## Gene Expression Data

We apply our tight cluster algorithm to a real data set on psoriasis^18^. This data set consists of microarray expression of 54675 probes for 38 case individuals suffering from psoriasis. These probes cover 17386 genes. The steps we take to analyse this data are as follows.

We run our algorithm for a specific number of tight clusters. After obtaining the clusters, we input the probes of each cluster to DAVID software^19^ to see how many functional annotations are covered by these probes. DAVID provides a classification of the probes into several biological processes or pathways; each process is indicated by a unique GO term. A group of such processes based on biological functionalities form a “functional annotation” or a “functional category”. Thus, the probes belonging to a functional category have some similarity as far as biological function is concerned. For example, one functional category may consist of positive regulation of different biological processes, each one being indicated by a unique GO term. We find how the probes in a tight cluster obtained by our algorithm are distributed to different such functional categories as obtained through DAVID. Note that, some probes representing a gene expression may also belong to more than one functional category whereas two different sets of probes may belong to the same functional category. Moreover, more than one probe may correspond to a single GO term.

**Figure 4:**
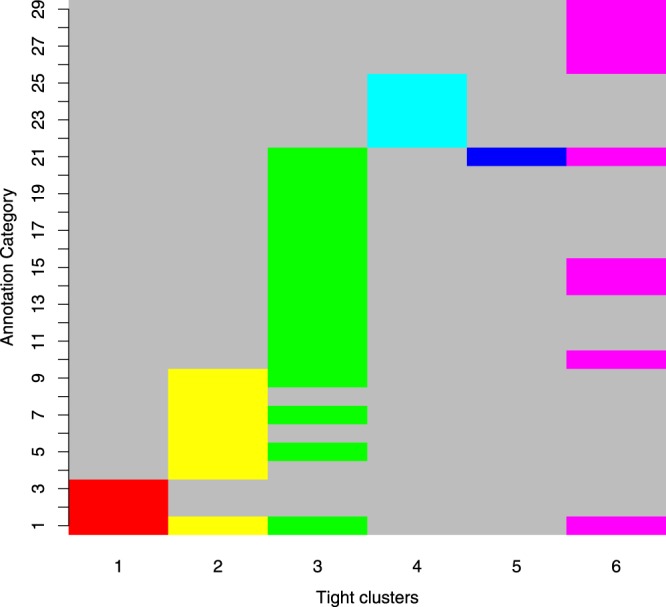
Functional annotations of the clusters are detected from tight clustering algorithm. Grey shade means the cluster has no representation in that category. Different colors are used for separate clusters. The non-overlapping block structures of the annotation categories for different clusters are clearly visible.

For the data set on psoriasis, we run our algorithm varying the number of target clusters from 4 through 10. We report the result when the target number of clusters is 6 in Fig. 4 (for 4 and 5, see Supplementary Figs S16 and S17). Our algorithm outputs 6 clusters with 2043, 3020, 8361, 2239, 3401, and 3718 probes respectively, out of which 44, 99, 205, 33, 59, and 66 do not belong to any functional category as shown by DAVID. The 31893 probes not included in any cluster are scattered over 76 functional categories and 674 processes (indicated by unique GO terms) not belonging to any functional category. The vertical axis in Fig. 4 represents 29 functional annotation categories identified from DAVID (see also Supplementary Figs S16 and S17). From this figure we see that probes in a tight cluster fall into one or more functional categories. We repeat this for each of the 6 tight clusters and check the amount of overlap among functional annotations for different tight clusters. As we see in Fig. 4, probes in the first tight cluster belong to 3 functional categories whereas probes in the second tight cluster belong to 7 such categories of which only 1 category is common between these two tight clusters. These overlaps are expected in real data, but the amount of such overlaps should be small. We see 6 distinct bands depicting the clusters. Hence, Fig. 4 clearly illustrates that the algorithm is successful in clustering probes in a large data set that has some similar biological functionality.

## Other Real Data Examples

The clustering algorithm developed in this article is tailored for data sets where clusters are expected to have variability in terms of their tightness. Gene expression clustering problem is of this nature. But we can still use tight clustering algorithm to other large data problems. In this section we discuss three more real data sets of different flavors to assess the performance of our algorithm.

The first data set we consider is Sensorless Drive Diagnosis data sourced from UCI machine learning data repository^20^, where features are recorded from electric motor current. The motor has different operating conditions that define the classes. Although the data consists of 48 variables, many of them are summary statistic of different measures. We have taken first 10 variables for our clustering problem and compared the output to the class labels. Sample size of this data set is 58509. We apply the proposed tight clustering algorithm with a target number of clusters 11. The Rand index is calculated to be 0.8374, ensuring that our algorithm works well in this data set.

The next two real data sets considered are on protein structures and its physicochemical properties. In these data sets there is no well-defined class labeling. We adopt different methods to assess the result of the proposed tight clustering algorithm.

The first data set, sourced from UCI machine learning data repository^20^, consists of measurements on 45730 decoys from protein structure prediction center (Critical Assessment of Structure Prediction 5 to 9 experiments). In Table 2 we denote this data set by CASP5-9. Each decoy has 9 measurements on physicochemical properties and the Root Mean Square Deviation (RMSD) measure on the quality of the predicted protein structure relative to the experimental structure. Another clustering problem relevant to these datasets is that of clustering 3D protein structures^21,22^. In this paper, we do not consider the clustering problem of protein structures. But, in future we hope to extend our algorithm in this direction.

**Table 2:** Results of the proposed tight clustering algorithm on various large size real data sets.

Data Set	n	Dimension	Details	Rand Index
Sensorless Drive Diagnosis	58509	10	Number of Clusters = 11	0.8374
CASP5-9	45730	9	Analysis (i)	0.805
			Analysis (ii)	0.8107 (0.79, 0.82)
			Analysis (iii)	0.7629 (0.7183, 0.7892)
CASP5-10 + RCSB	99987	6	Analysis (i)	0.895
			Analysis (ii)	0.8358 (0.8087, 0.8693)
			Analysis (iii)	0.9171 (0.7807, 0.9289)

For each of the two Protein Structure and Physicochemical Properties data sets three analysis and corresponding three Rand index values are presented. Three analyses: (i) Comparing *K*-means clustering results on the protein prediction quality, i.e., RMSD, and result of proposed tight clustering on physicochemical measurements; (ii) Comparing result of *K*-means clustering on 1/10th the data set and class labeling of the same 1/10th data set calculated based on result of the proposed tight clustering on the 9/10th of the data set; (iii) Comparing result of tight clustering on 1/10th the data set and class labeling of the same 1/10th data set calculated based on result of the proposed tight clustering on the 9/10th of the data set. For (ii) and (iii) we report the median, with the first and the third quartiles in the parenthesis, of Rand indices over 10 parts.

In the second data set, the modeled structures are taken from protein structure prediction center (CASP-5 to CASP-10 experiments), public decoys database and native structure from protein data bank (RCSB)^23^. In Table 2 we denote this data set by CASP5-10 + RCSB. There is a total of 95091 modeled structures of 4896 native targets. A total of 99987 instances with 6 multiple physicochemical measurements along with the RMSD of modeled protein structure in the absence of its true native state is available.

We evaluate the performance of our clustering method on these data sets by using following two approaches. In the first approach, we apply our proposed tight clustering method on the physicochemical properties and apply *K*-means algorithm on the quality measure and compare the two clusterings using Rand index. This comparison makes sense because of the following argument. It is known that, physicochemical properties of amino acid essentially generate various types of energy contributors such as electrostatic, van der Waals, salvation/desolvation which create folding pathways of a protein sequence into its unique tertiary structure^24^. When the structure is not known, homology modeling uses experimentally known protein structures as templates and based on amino acid sequence similarity, predicts the protein structure^25^. The structures so determined, after passing quality assessment, usually look similar to the ‘target’ structure but could indeed have large distance at a high resolution^26^. This deviation contributes to the RMSD values. Since homology modeling only uses sequence information, deviation is likely determined by the amino acid sequence. Now, as noted above, in biology, physicochemical properties essentially determine the sequence. Thus, similar physicochemical properties of amino acid are expected to have similar patterns of deviation, i.e., similar RMSD values. Indeed, if this relationship can be understood, that would be a big step forward in accurate prediction of high-resolution protein structure. For now, we are only interested in whether our algorithm can recognize this relationship.

But, the difficulty we face here is that the number of clusters is unknown. To resolve this problem, on the variable RMSD, we use the gap statistic^8^ with *K*-means algorithm to determine the number of clusters. For the first data set the gap statistic determines 17 clusters while for the second data set it determines 40 clusters on the quality of protein structure prediction. Next to apply the tight clustering algorithm on the physicochemical properties we use the target number of clusters as 17 and 40 respectively. But the tight clustering algorithm only finds 11 and 36 clusters respectively for the two data sets. The Rand index values are 0.805 for the CASP5-9 data set on 45730 decoys and 0.895 for the CASP5-10 + RCSB data set on 99987 protein structures. The results are presented in Table 2 under label ‘Analysis (i)’.

In the second approach, first we ignore the quality information altogether and only consider the physicochemical properties. We use a cross-validation method to assess the performance of the clustering method. We randomly partition the data into 10 parts. First fix one part and run our tight clustering algorithm on remaining the 9/10th of the data. Based on the resulting clusters, we label the 1/10th data. Now we apply *K*-means on the 1/10th data again and compare these two sets of labels. Repeat the procedure for all the 10 parts of data, each one at a time. We again repeat the same procedure using our tight clustering method instead of *K*-means and compare the two sets of labels. We present summary statistics of 10 Rand indices obtained for both *K*-means and tight clustering in Table 2 under labels ‘Analysis (ii)’ and ‘Analysis (iii)’ respectively. The Rand index for the two data sets are 0.8107 and 0.8358 (for Analysis (ii)); 0.7629 and 0.9171 (for Analysis (iii)) respectively.

## Discussion

The importance of large data clustering algorithms in empirical sciences were realised as early as in the 90’s. Extension of PAM algorithm for large data to propose CLARA^3^ and later CLARANS^27^ laid an impressive track. The concept of large data has changed quite a bit since then, credited mostly to the involvement and advancement of technology. The analysis of large data sets requires efficient and informative decision making in minimum time.

In this paper we extend the utility of tight clustering method to large data that in some parts mimic the original tight clustering ideology. Here the stable and tight clusters are picked up in a sequential manner that is one by one iteratively preserving the central idea of the basic tight clustering method.

The fundamental strategy behind our proposed algorithm is initially dividing the data and then, pull the learnings together from the divided parts. While combining information from smaller parts, we used an operator ${\mathbb{C}}$ that makes use of a choice function *π*. In the final step of the algorithm, to extract out the most reliable information, we use a measure *σ*. The guideline for practical choice of the functions *π* and *σ* are also provided. We show that in the ideal situation the probability of concordance goes to 1 as we increase the number of repetitions. However, the number of such repetitions is not very large in practical implementation; our experience based on extensive simulation indicates that value of 3 is enough to get reliable clusters.

Tight clustering algorithm looks specifically for noise. So, noisy data are more useful to tune the clusters than data without noise. This feature can be used to get better practical results by artificially imputing noise in higher dimension when it is known in advance that there is not enough noise. Thus, noise level can be treated as a pseudo tuning parameter for the proposed algorithm in the sense that rather than adjusting parameters for the original tight cluster algorithm calls inside our proposed algorithm one can impute noise and use the default parameters to get better results. We call this feature “noise tuning.”

### Run time

We also considered a critical analysis of the run time of our proposed algorithm. Based on the run time results for the sub-routines we have established that theoretically run time of the proposed algorithm is no worse than the original tight clustering algorithm. Implementation of tight clustering algorithm in R package ‘tightClust’ is unable to incorporate more than 3000 genes. Table 3 represents run times for 10- and 20-dimensional data with sample size as 10000 and 20000, for noise percentage as 5%, 10%, 15%, and 20%. This program runs in a Mac (OS X, version 10.9.5) computer with 3.5 GHz 6-core Intel Xeon E5 processor having 16 GB RAM, directly in an external USB connected hard drive. Thus, our proposed algorithm is computationally tractable for large data sets. Our code written in R is freely available upon request.

**Table 3:** Average run time (in minutes) based on 10 replications for our tight cluster algorithm; d = dimension, SS = sample size.

d	SS = 10000		SS = 20000	
	10	20	10	20
noise				
5%	6.07	7.32	12.90	16.72
10%	5.95	6.97	13.68	20.70
15%	6.05	7.90	13.90	19.55
20%	7.11	7.81	13.74	21.81

### Parallel computing

The proposed version of the tight-clustering algorithm for large data sets can easily be implemented in a parallel computing infrastructure. Both the steps of the algorithm, partitioning and combining, can be applied in a parallel manner. First, the partitioning step runs *R*(*n*) independent processes on the same data. Thus, partitioning step can be implemented in *R*(*n*) machines that use a shared storage system containing the data. Second, in the next step of the algorithm, combining the output clusterings of the partitioning step: while it cannot be completely implemented in a parallel manner, we can partially parallelise the job. The combining processes can be thought of as a left fold operation where the binary operator involved combines two sets of clustering outputs. This folding operation can be carried out in a parallel manner by considering a bunch of sub-jobs which combines exclusive subsets of all the clusterings from the partitioning step. Finally, the output of these sub-jobs can be processed. We expect a substantial improvement in runtime through this parallel processing.

## Conclusion

Many statistical methods developed and studied for data sets of smaller size and dimension must find their footing in large scale problems in science we frequently face today. This paper considers one such classical problem in statistics, clustering, which has become a helpful companion to research in many fields and proposes a scalable algorithm useful for large datasets. In recent years the scope of clustering method has been expanded by various field specific algorithms, see e.g.^28,29^, for couple of recent entries to this list. Our proposed clustering algorithm is tailored for analysis of microarray gene expression where a clustering algorithm often is the focus tool of analysis^30–34^; however, it can easily be applied to any other large dataset generated from experiments other than microarray, e.g. RNA-seq, scRNA-seq, etc. We hope our proposal will be a worthwhile entry to the literature. We have vetted our algorithm theoretically, using simulation, and by real data sets. The algorithm has been made available to the researchers via easily implementable R code^35^. The code can be obtained upon request or directly downloaded from the link: same https://github.com/sarmistha123/TightClust_Large/blob/master/TightCluster_Large.R.

## Supplementary information

Supplementary Material
