# Supplementary material for: Tight clustering for large datasets with an application to gene expression data
Source: Sci Rep. 2019 Feb 28;9:3053. doi: 10.1038/s41598-019-39459-w (PMC6395712; doi:10.1038/s41598-019-39459-w)
Supplement: Supplementary file 1 — Supplementary Material [file 41598_2019_39459_MOESM1_ESM.pdf]

Supplement : Tight clustering for large datasets with an application to gene expression data

Bikram Karmakar[1], Sarmistha Das[2], Sohom Bhattacharya[2], Rohan Sarkar[2], and Indranil Mukhopadhyay[2,*]

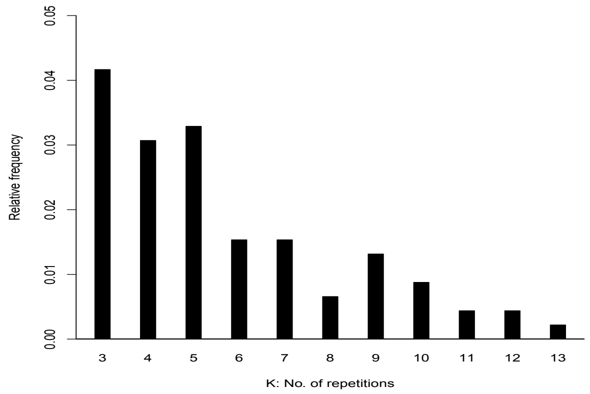

Figure S1: Frequency distribution of maximum two Rand Indices over repetitions.

[1] Department of Statistics, University of Pennsylvania, Philadelphia, U.S.A.
[2]Human Genetics Unit, Indian Statistical Institute, Kolkata, India
* email : indranil@isical.ac.in

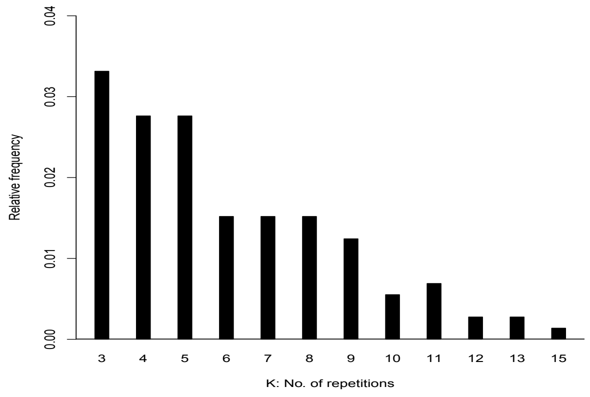

Figure S2: Frequency distribution of maximum three Rand Indices over repetitions.

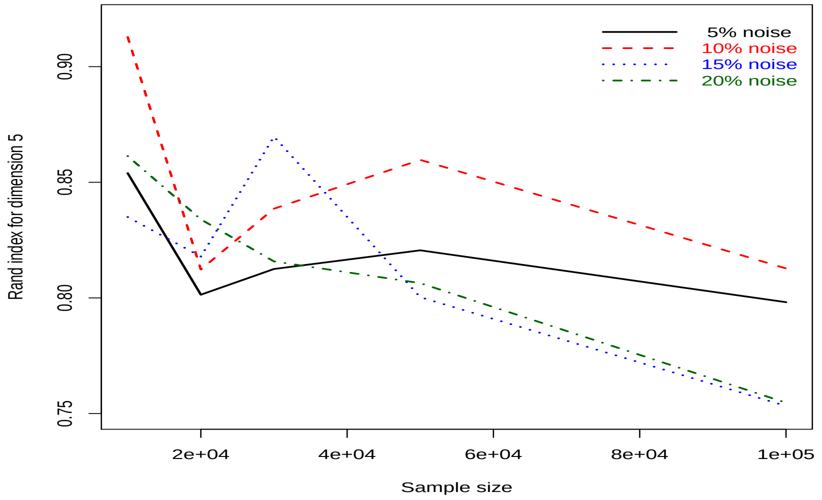

Figure S3: Rand Index plot for diffferent sample sizes varied over noise levels for fixed dimension 5.

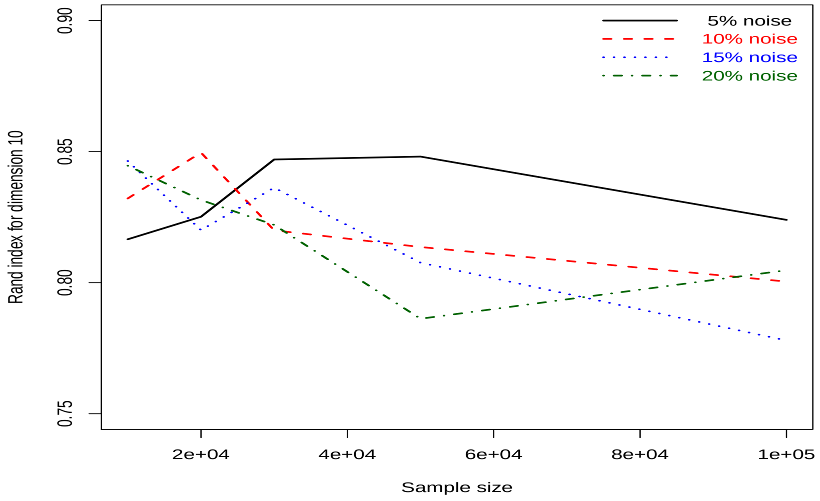

Figure S4: Rand Index plot for diffferent sample sizes varied over noise levels for fixed dimension 10.

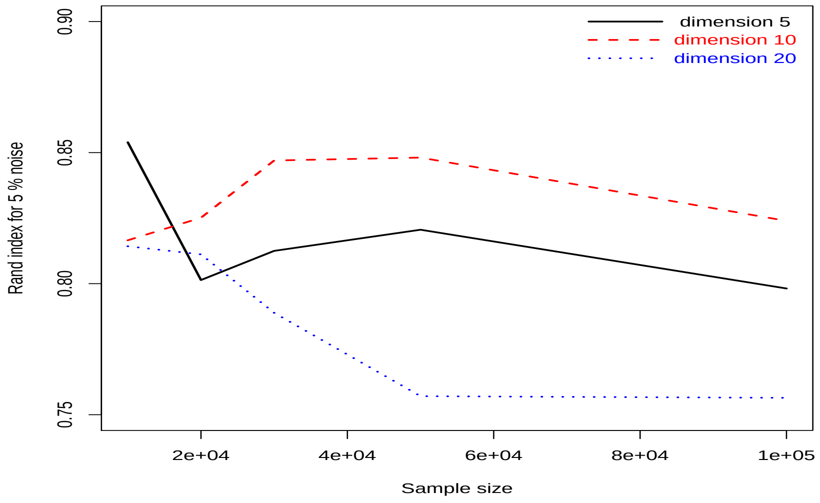

Figure S5: Rand Index plot for diffferent sample sizes varied over dimension for noise level 5%.

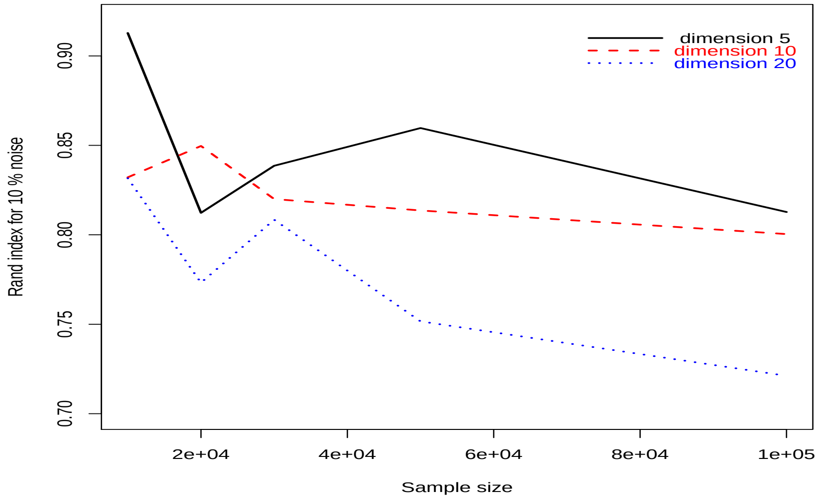

Figure S6: Rand Index plot for diffferent sample sizes varied over dimension for noise level 10%.

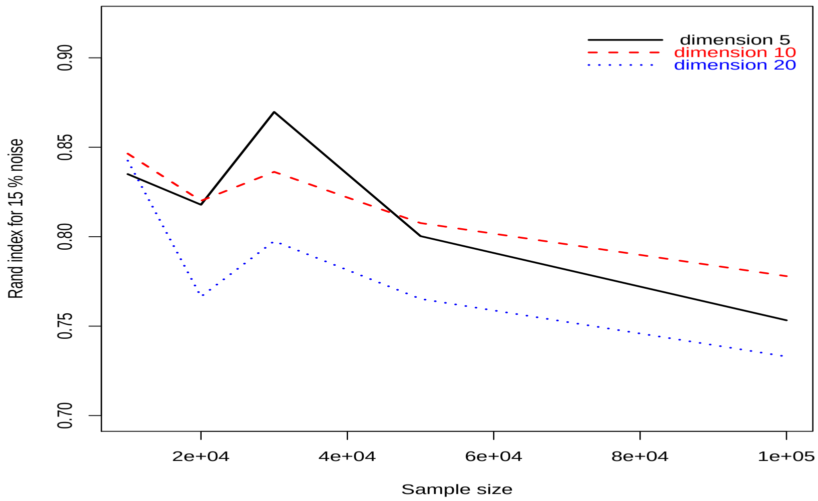

Figure S7: Rand Index plot for diffferent sample sizes varied over dimension for noise level 15%.

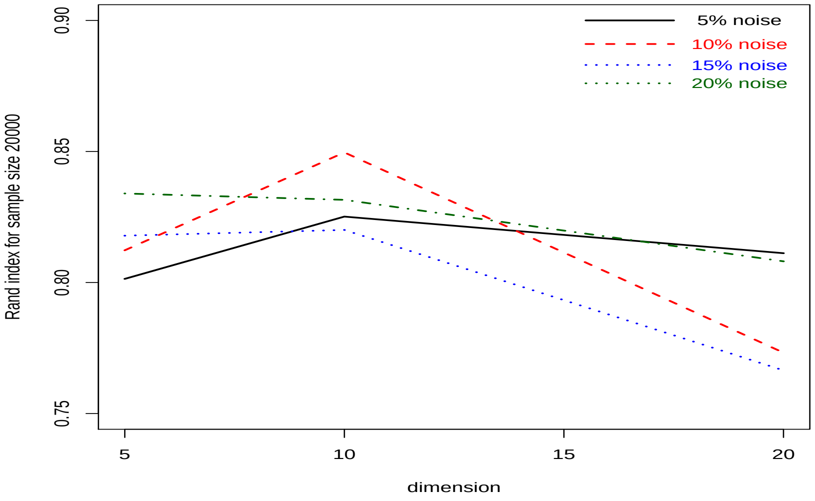

Figure S8: Rand Index plot for diffferent dimensions varied over noise levels for sample size 20000.

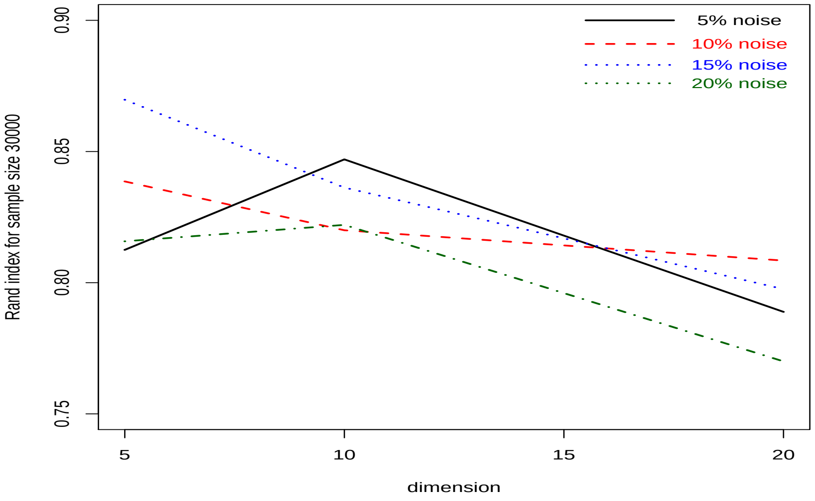

Figure S9: Rand Index plot for diffferent dimensions varied over noise levels for sample size 30000.

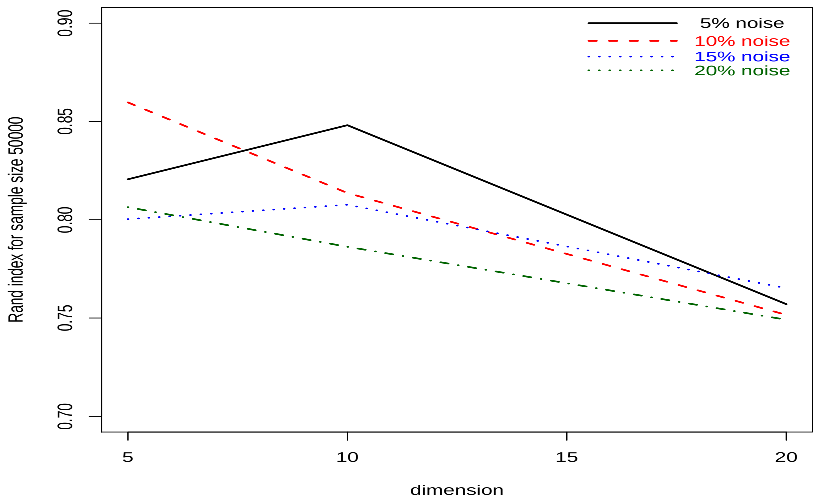

Figure S10: Rand Index plot for diffferent dimensions varied over noise levels for sample size 50000.

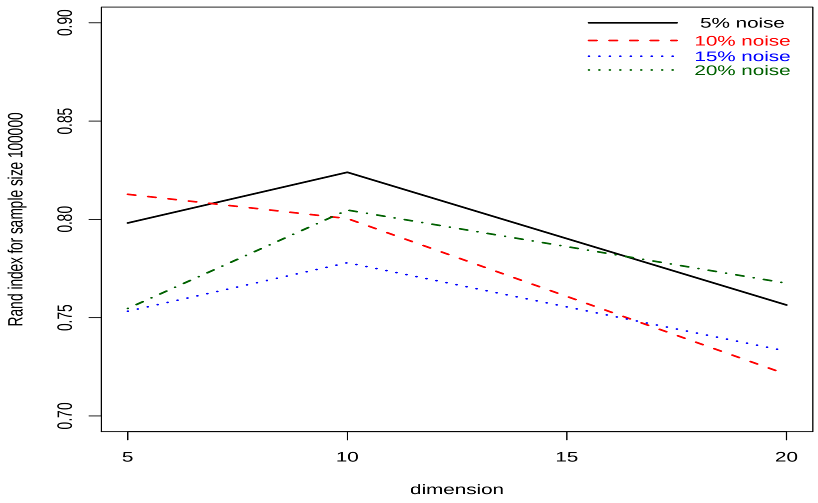

Figure S11: Rand Index plot for diffferent dimensions varied over noise levels for sample size 100000.

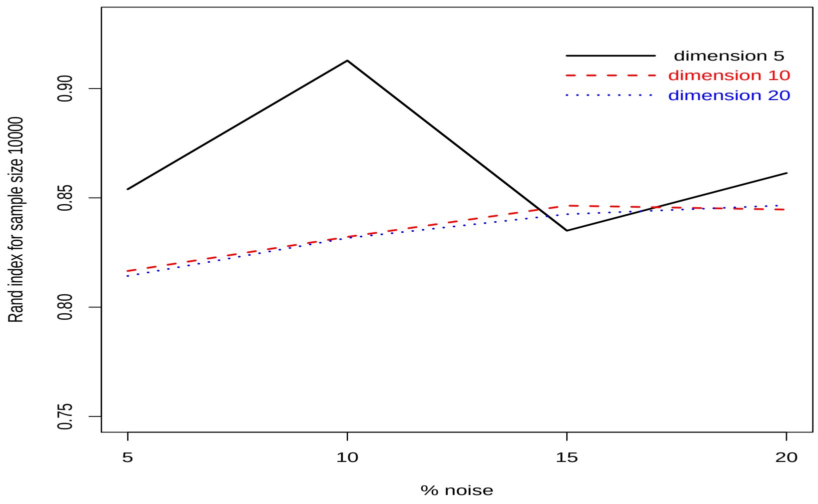

Figure S12: Rand Index plot for diffferent noise levels varied over dimensions for sample size 10000.

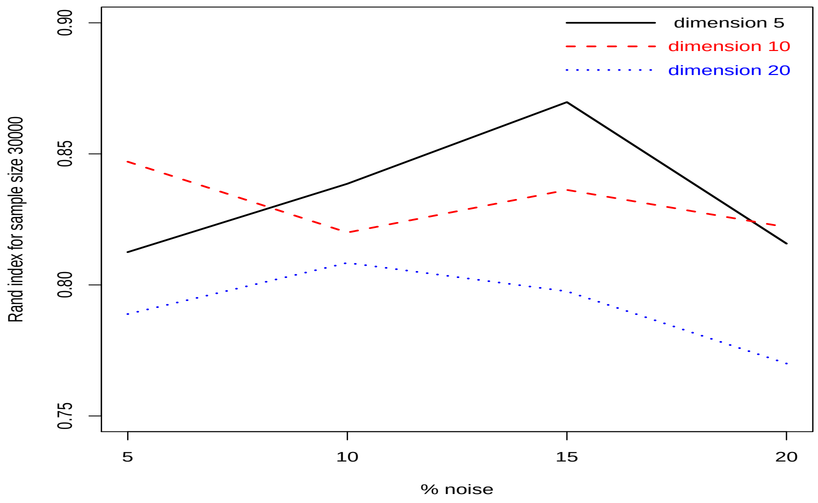

Figure S13: Rand Index plot for diffferent noise levels varied over dimensions for sample size 30000.

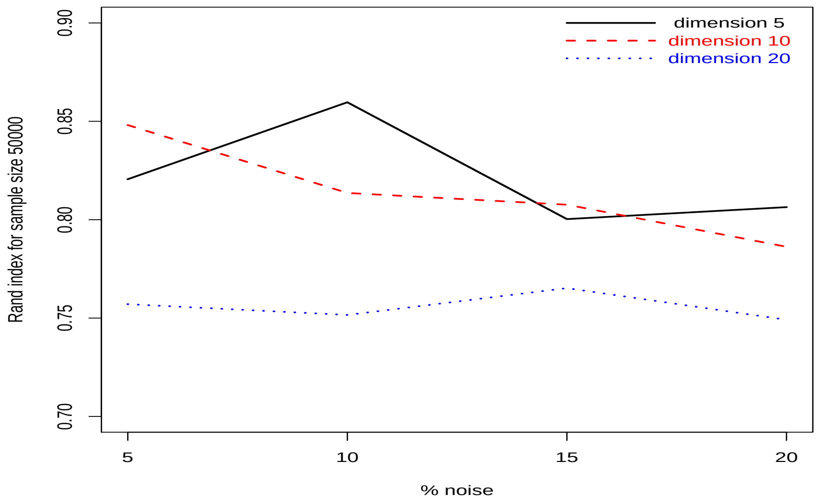

Figure S14: Rand Index plot for diffferent noise levels varied over dimensions for sample size 50000.

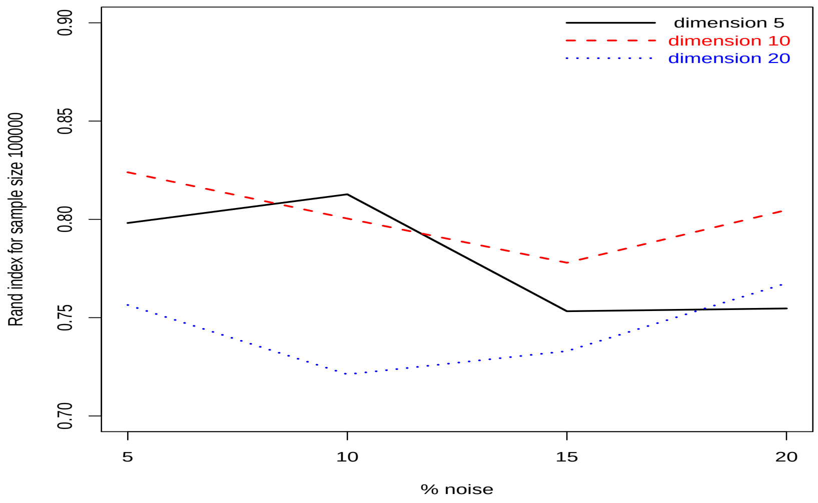

Figure S15: Rand Index plot for diffferent noise levels varied over dimensions for sample size 100000.

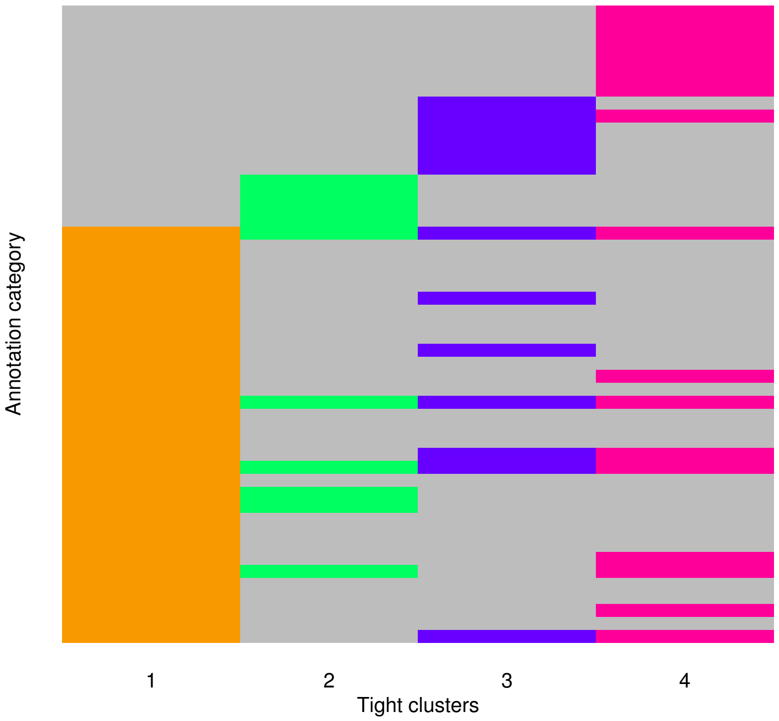

Figure S16: Functional annotations of the clusters detected from tight clustering algorithm (with k = 4).

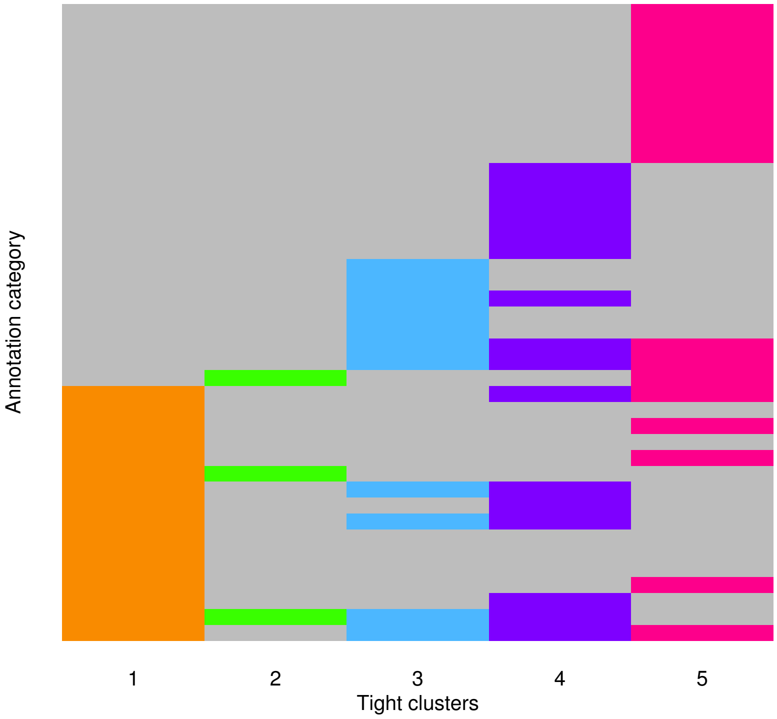

Figure S17: Functional annotations of the clusters detected from tight clustering algorithm (with k = 5).
